# Hotspot generation for unique identification with nanomaterials

**DOI:** 10.1038/s41598-020-79644-w

**Published:** 2021-01-15

**Authors:** Nema M. Abdelazim, Matthew J. Fong, Thomas McGrath, Christopher S. Woodhead, Furat Al-Saymari, Ibrahim E. Bagci, Alex T. Jones, Xintai Wang, Robert J. Young

**Affiliations:** 1 Department of Physics, Lancaster University, Bailrigg, LA1 4YB UK; 2 School of Electronic and Computer Science, University of Southampton, Southampton, SO17 1BJ UK; 3 School of Computing and Communications, Lancaster University, Bailrigg, LA1 4WA UK; 4 Cavendish Laboratory, University of Cambridge, J J Thomson Avenue, Cambridge, CB3 0HE UK

**Keywords:** Nanoparticles, Optoelectronic devices and components, Nanophotonics and plasmonics, Nanoparticles, Quantum dots

## Abstract

Nanoscale variations in the structure and composition of an object are an enticing basis for verifying its identity, due to the physical complexity of attempting to reproduce such a system. The biggest practical challenge for nanoscale authentication lies in producing a system that can be assessed with a facile measurement. Here, a system is presented in which InP/ZnS quantum dots (QDs) are randomly distributed on a surface of an aluminium-coated substrate with gold nanoparticles (Au NPs). Variations in the local arrangement of the QDs and NPs is shown to lead to interactions between them, which can suppress or enhance fluorescence from the QDs. This position-dependent interaction can be mapped, allowing intensity, emission dynamics, and/or wavelength variations to be used to uniquely identify a specific sample at the nanoscale with a far-field optical measurement. This demonstration could pave the way to producing robust anti-counterfeiting devices.

## Introduction

Physically Unclonable Functions (PUFs) are a form of hardware cryptographic primitive, that allows for the authentication and identification of physical objects^1^. When applied towards the authentication of electronic devices, this authentication usually occurs through entirely electronic channels, but when physical objects are to be verified, optical evaluation methods are typically more practical^2,3^. A valuable metric for a PUF is the number of unique challenge-response pairs (CRPs) a potential device can provide. Increasing the number of CRPs supported by a device has a variety of benefits, including the ability to concatenate the CRPs to increase the total response length. This increase in the response length enhances the level of security of a single exchange. Other benefits include reducing the error rate by introducing sacrificial bits for post-processing, or the ability to separate responses to reduce vulnerability to replay attacks^4,5^.

Naturally, the number of useful extractable bits from a given device is directly related to the range of potential analogue values that a measurement can take before digitisation. Therefore, to increase the number of available CRPs, either the resolution of the measurement or the dynamic range of the measured parameter of the PUF can be increased. The former often comes with additional requirements, and so it is this second avenue of development for a quantum dot PUF (QD-PUF) that is presented in this paper.

The QD-PUF consists of colloidal quantum dots, distributed on a surface in a manner which is random and uncontrollable during the fabrication process^6^. When the sample is illuminated above the bandgap of the dots (by a laser or otherwise), they emit photoluminescence (PL), which can then be measured, digitised, and converted into a unique fingerprint whose uniqueness originates from the random spatial distribution of the QDs^7^. Typically, the smaller the type of particle to be deposited on a surface, the harder the corresponding PUF is to clone. This is due to the increase in precision needed when manipulating or emulating the individual particles; size and composition fluctuations also lead to greater variations in emission properties^8,9^.

This paper seeks to probe the efficacy of adding gold plasmonic nanoparticles (Au NPs), in conjunction with a reflective sample back-coating, in the form of a thin layer of aluminium (Al), to improve the dynamic range of emission intensities of a random distribution of QDs. In this case, heavy-metal free InP/ZnS core/shell QDs are examined due to their low toxicity, low environmental cost, high adsorption coefficient and desirable emission wavelength for use with silicon-based sensors^10^. This makes them an attractive candidate for practical deployment, as part of an optical authentication device. Au NPs were transferred from solution to the substrate via a very simple dropcasting method, increasing the feasibility of including nanoparticle treatment in the fabrication process. Previous studies on the deposition of colloidal metal nanocrystals on a variety of substrates have mainly focused on electrostatic deposition, changing the chemical treatment of the substrate, spray deposition and Langmuir–Blodgett technique^11,12^. These methods are generally limited with non-uniform particle densities, changing functional groups or surface charge that need multiple preparation steps, or can only cover small areas. By controlling the concentration of NPs in the solutions used for preparation, their distribution and density can be tuned. This method offers a fast and simple procedure compared to other currently known metal nanocrystals deposition techniques. After fabrication, optical microscopy was used to show that these large-area plasmonic structures are highly sensitive to their relative positions, causing the formation of localised emission enhancement (hotspots). PL maps were taken on several marked areas based on atomic force microscopy (AFM) and scanning electron microscopy (SEM) techniques, in order to obtain quantitative information about the bright PL spots. The AFM and SEM techniques examine the spatial extent of the Au NPs on the marked map areas, and the micro-PL (μPL) system scans the sample’s surface, collecting PL spectra data at each point and mapping variations in the intensity, wavelength and width of the measured PL signal.

## Methods

Two different samples were fabricated to observe the effect of plasmonic nanostructures on the spatial distribution of PL.

**Figure 1:**
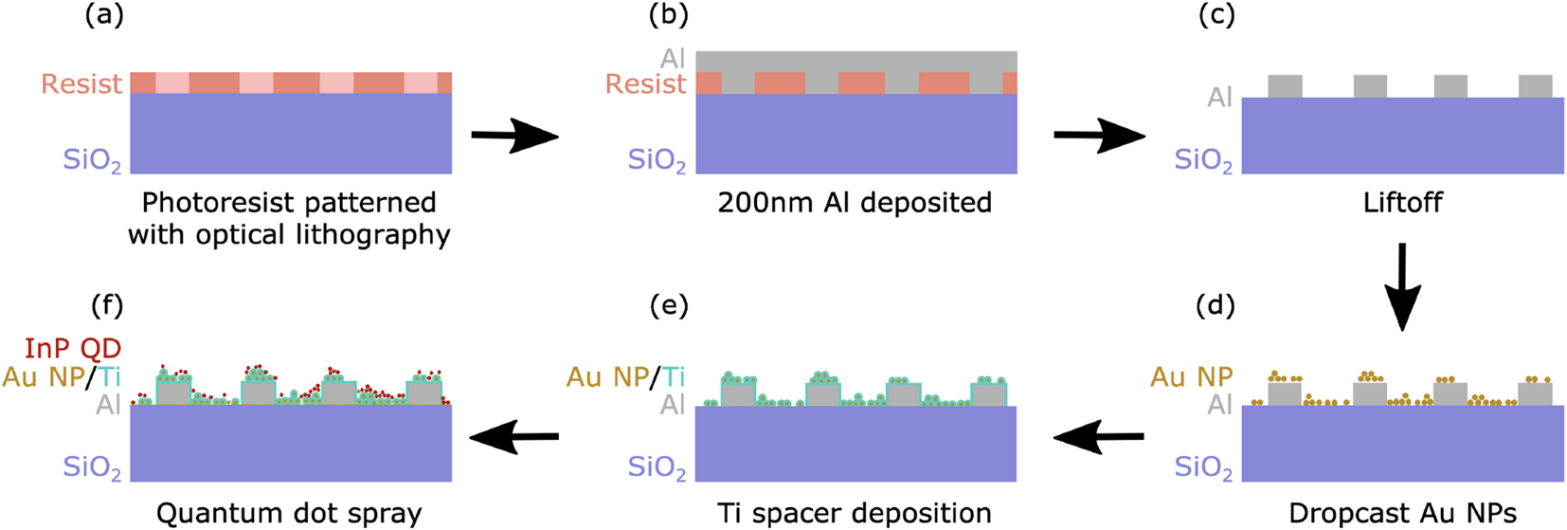
A schematic view of the fabrication step process of the plasmonic nanostructure sample using photolithography. Fabrication procedures: (**a**) Photoresist was spin-coated on the substrate, and the mask patterns were aligned for UV exposure. (**b**) Al layer was deposited using a thermal evaporator. (**c**) Lift-off process to leave Al grids (**d**) Dropcast Au NPs, (**e**) TiO_2_ spacer layer deposition using e-beam evaporation. (**f**) Electrospray of colloidal InP/ZnS QDs.

Sample A was a control sample and sample B contained plasmonic NPs and an additional, 200 nm thick, base layer of Al on the substrate, which was patterned into rectangles, before depositing the NPs. Figure 1 shows a schematic of the fabrication method of the plasmonic sample. The presence of the Al array serves two purposes: it creates reference points, so specific regions of interest on the sample can be easily located, and it also enhances the absorption and external efficiency of the system, with the highly reflective surface increasing the coupling efficiency into and out from the QDs.

It has previously been shown that direct contact of a metal with fluorescing materials leads to a strong quenching effect, due to the introduction of a very efficient non-radiative recombination channel^13–16^. Therefore, a thin layer of dielectric (TiO_2_) was deposited onto the Au NP substrate, to separate the QDs from the metal nanoparticles. The thickness of the dielectric was optimised to create the best coupling between NPs and QDs. See Supporting Information Sect. 1 for more details. The thin TiO_2_ layer has a negligible impact on the overall substrate reflectivity, with a transmittance of > 90%, and reflectance < 10% in the visible range. The reflectivity of the SiO_2_ layer has been investigated previously^17^, where it was found that the reflectivity of the Al structure is over 2× higher than the sample with just 300 nm SiO_2_^18^. This enhances the total external efficiency by propagating more of the emitted light in a useful direction and incident light back towards the QDs.

**Figure 2:**
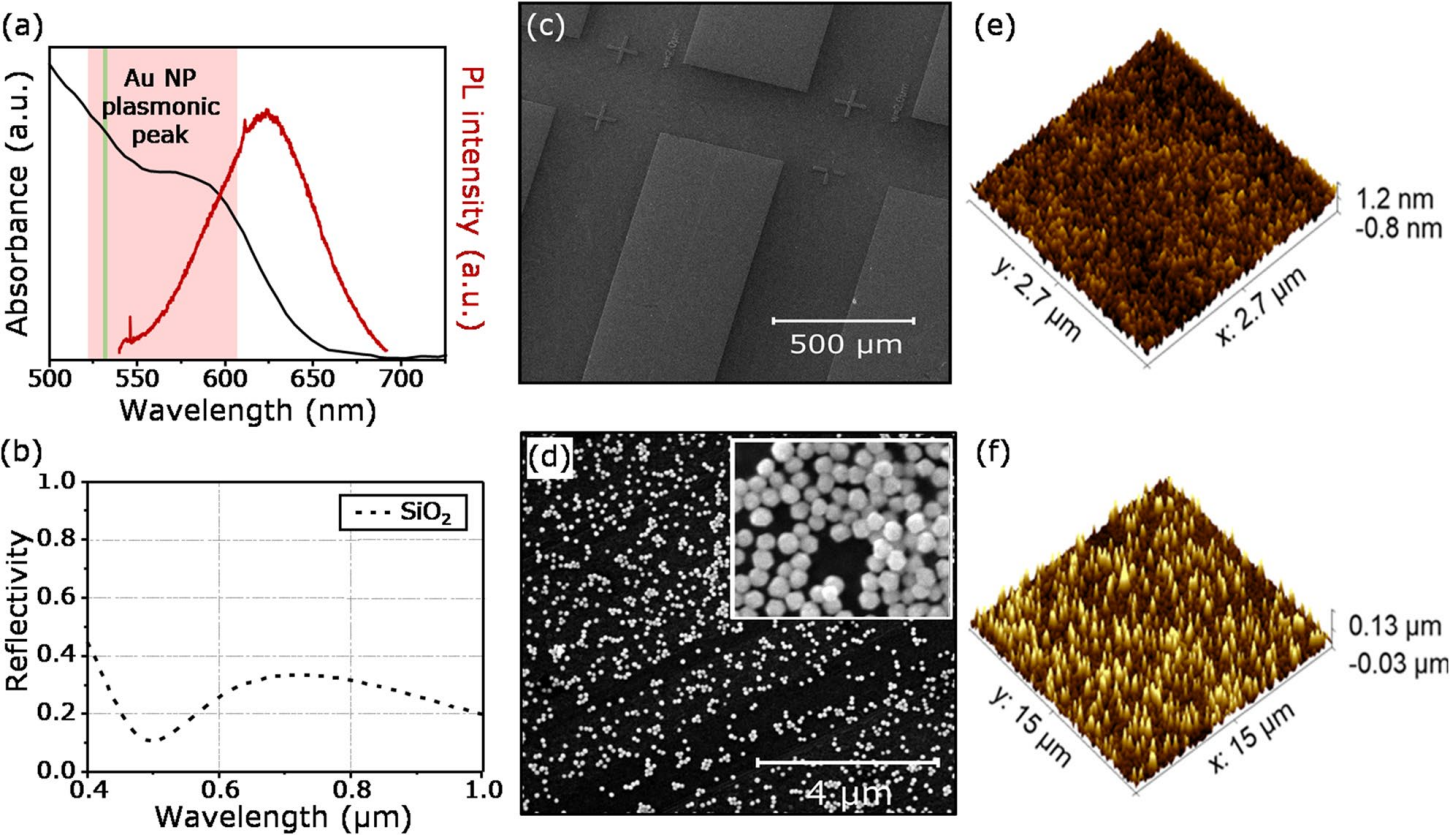
(**a**) Absorption spectra and photoluminescence (PL) spectra of InP/ZnS QDs film. The laser wavelength used is 532 nm (green vertical line). The highlighted red area corresponding to plasmon resonance peak of the Au NPs. (**b**) Reflectivity of thick layer SiO_2_. (**c**,**d**) SEM images of Al pattern and Au NPs on top of Al using the dropcast method, inset showing high-resolution SEM image. (**e**,**f**) 3D AFM images of the QDs without (control) and with Au NPs on top of the Al nanostructure sample taken at location from samples A (**e**) and B (**f**).

A suspension of Au NPs with an average radius of 100 nm, with a concentration optimised to give a uniform distribution when deposited, was dropcast onto the surface of sample B. These Au NPs have a peak plasmon absorption wavelength of 575 nm. This optimised dispersion of NPs was spread uniformly on the surface of the substrate, with a dense but well-separated distribution, with an average coverage of 105–110 μm^−2^. SEM and AFM images of the plasmonic nanostructure samples are shown in Fig. 2. Supporting Information Sect. 2 contains more details about the optimisation of the Au NP solution concentration. The QD solution was deposited onto the substrates using electrospray, with both the control and plasmonic samples being prepared simultaneously. This technique projects a fine spray of a polar solution onto a substrate. When optimised, the spray ensures the solvent evaporates before reaching the surface, preventing QD clustering on the surface (See Supporting Information Sect. 3 for more details). This is important because any QD clustering will reduce the likelihood that they will be able to be deposited in the gaps between the Au NPs.

The measured absorption and emission spectra of the QD films are shown in Fig. 2a. The maximum PL peak intensity of the films is centred at 620 nm, whilst the absorption peak is 580 nm. The absorption peak of the plasmonic nanoparticles ranges from 520 nm and extends to longer than 600 nm. It is centred at 575 nm, which overlaps with the absorption and emission spectra of the QDs, leading to the possibility of local electric enhancement^19,20^.

## Results

### Examination of PL hotspots

**Figure 3:**
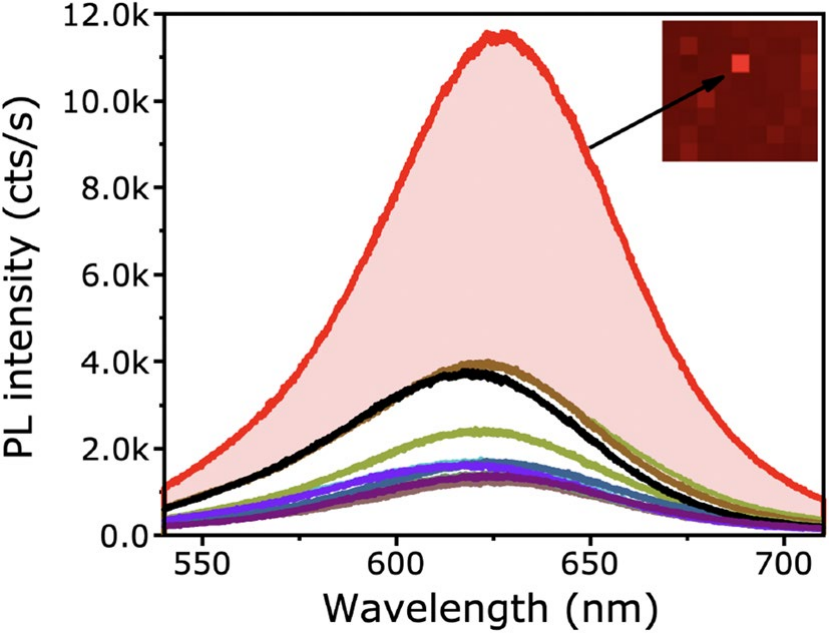
Representative set of PL emission obtained from different positions on the surface of the plasmonic nanostructure sample, with 5 μm steps at the selected marked area (B1). Inset: Mapping distribution of the same area, showing bright hotspots caused by plasmonic resonant enhancement from the Au NPs.

Figure 3 shows PL spectra, taken across the plasmonic sample at area B1 with 5 μm steps. This map was performed over the same area as the AFM image shown in Fig. 2f, verifying the presence of NPs. A sixfold enhancement in the average PL intensity is observed at the maximum coupling between the NPs and the QDs. The area of maximum coupling can be seen as bright spots in the map. The control sample (A) has a PL intensity of around 1500 cts/s (see Supporting Information Sect. 4), where the maximum of the sample with Al and NPs is around 11,000 cts/s. There is also a slight narrowing of the peak from 80 nm at full-width at half-maximum (FWHM) for the control to 70 nm for the samples containing NPs, which indicates coupling between the NPs and the QDs. The μPL measurements were taken with a 532 nm laser with around a 1 μm spot size. The measurements were taken at 3.5 μm steps to avoid excessive crosstalk between measurements. When focused on the surface, the Airy disks produced will capture information from areas far beyond the extent of the laser spot, so we aimed to reduce degeneracy in our measurements.

**Figure 4:**
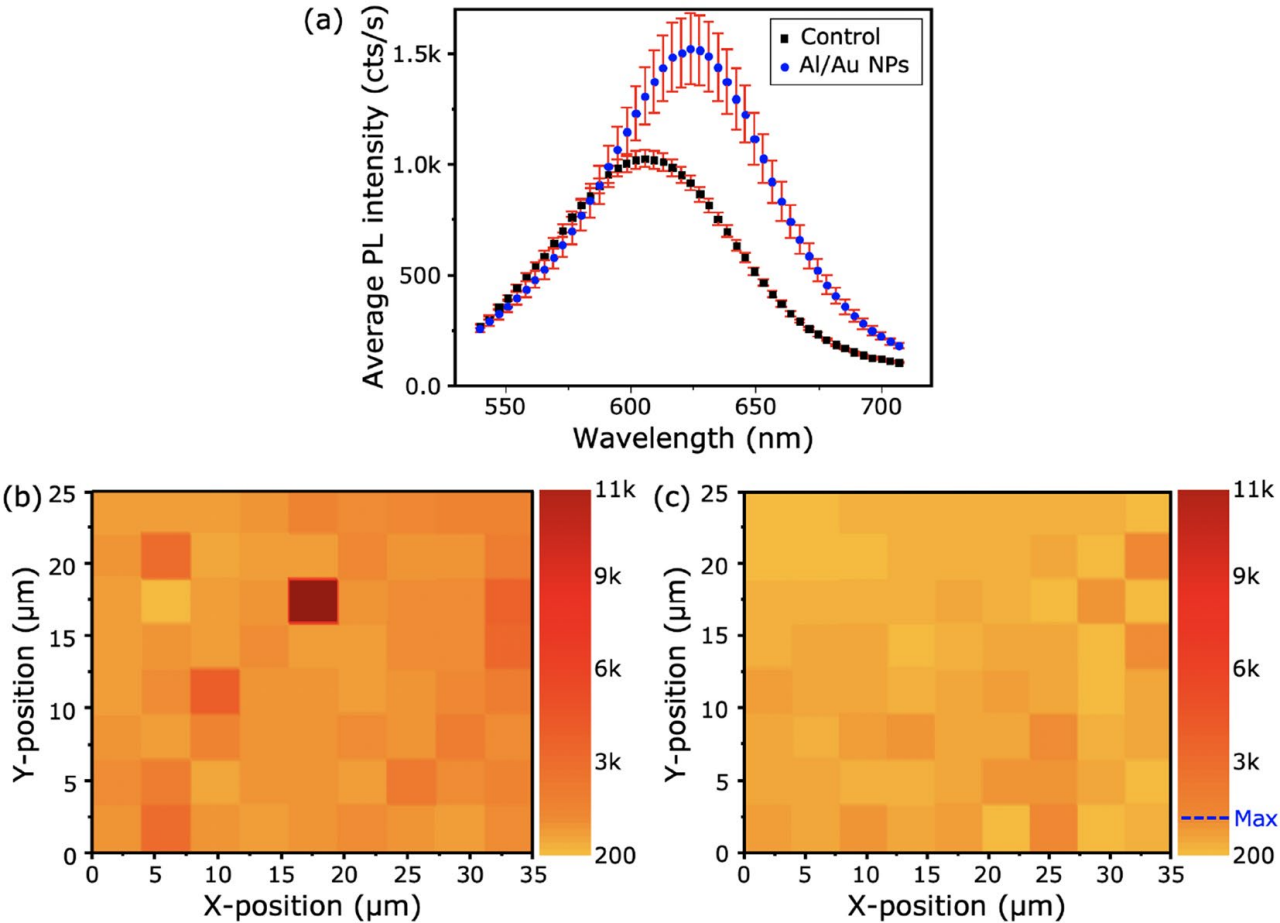
(**a**) Average PL measurements for control (A) and plasmonic nanostructure (B) samples were performed in a marked scanning area (A1 and B1). PL mapping intensity distribution of the plasmonic structure (**b**) and the control (**c**) samples at same location from where the PL measurements were taken with the colour scale representing PL peak intensity.

To ensure the density of both the control and plasmonic samples were comparable, they were sprayed simultaneously. Further discussion of density optimisation and procedure is detailed in Supporting Information Sect. 3. We can demonstrate the dramatic increase in the dynamic range of intensities from the control sample to the plasmonic nanostructure sample, by comparing the standard deviation. The average standard deviation of all intensity measurements taken over the control sample is σ = 260 cts/s, in comparison to the plasmonic sample, where it is σ = 3360 cts/s. This dramatic increase in standard deviation, alongside AFM and optical microscopy gives us confidence in attributing the bright spots to interactions with the AuNPs. From this, the hotspot enhancement can be attributed to the plasmonic interactions between QDs and NPs, and not any other factor, such as QD aggregation, which could conversely lead to quenching of the PL^21,22^. A set of PL spectra from the control sample is shown in Supporting Information Sect. 4.

A slight redshift of 15–20 nm in the PL peak was observed in the plasmonic sample. This shift is attributed to variations in the local dielectric constant for the NPs + TiO_2_, which could arise from slight thickness variations when depositing a very thin layer of metal using this method^23–25^. The plasmon resonance also varies and enhances the local electric field, causing a Stark shift.

A map of PL emission was taken of sample A, in which the distribution of intensities over this sample were observed to be highly uniform, which is in sharp contrast to the maps taken over the plasmonic samples. Notably in sample B, a much wider dynamic range of emission intensities was seen, including intense hotspots.

It is of note for this application that the NPs significantly impact the PL intensity distribution and line shape of the emission, relative to that of the control sample. There are observations in the plasmonic sample of not only hotspots and enhancement, but there is evidence of quenching of the PL. This quenching is attributed to aggregation of the NPs on the surface of the samples, which is supported by AFM and SEM images, and also variation in thickness of the dielectric layer, which is discussed further in Supporting Information Sect. 5. As discussed earlier, any variation in thickness of the dielectric will have strong implications for the overall enhancement factor of the QD emission.

### Hotspot randomness

**Figure 5:**
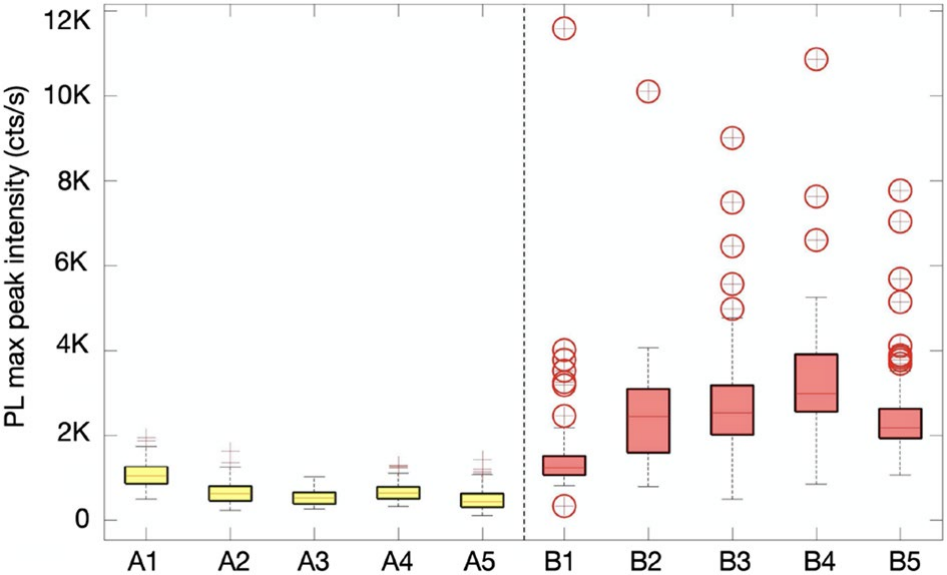
Boxplot showing the distribution of the PL maximum peak intensity for two groups at five marked different areas. PL measurements were performed on control sample A and plasmonic sample B. Each marked point has an area of 25 × 35 μm. Red circles refer to abnormal PL intensity values, which indicate the presence of the Au NPs.

Quantitative analysis of the PL maps was used to obtain useful information about the PL hotspot/quenching distribution and overall randomness in the intensity distribution across all samples. Figure 5 is a boxplot of the two QD sets, detailing the distribution of PL intensity from 5 maps taken across different areas of each sample. The plot shows the distribution of maximum PL intensity vectors for each map, including minimum, upper/lower quartile, median, maximum, and outliers. The red scatter symbols represent the presence of the Au NPs, which are clearly increased in the plasmonic sample with the existence of hotspots.

Different peak maxima were observed for each mapping area. This variation arises from several factors: firstly, the local density of QDs on the surface will create some spatial variation in emission intensity. This result is to be expected from the simple fabrication procedure and is present in both the plasmonic sample and the control sample. Additionally, the unpredictable and uncontrollable distribution of NPs on the surface, like the QDs, creates spatial variation in distribution, which then varies the local electric field on the sample.

When the inter-particle spacing is less than the particle diameter, there is a strong increase in local electric field, which decreases as the spacing decreases^26–30^. The observed reduction in hotspot intensity is caused by a decreased plasmon coupling across the gap.

The plasmonic sample’s emission hotspots are significantly enriched with the addition of the Al. This is caused by the increased surface scattering from the Al layer^31,32^. The NPs couple with their mirror image in the metallic film, enhancing the electromagnetic field at the junction^32^.

### Practical considerations and future work

Whilst practical implementation methods have been discussed elsewhere in the literature, we feel it is important to briefly discuss how our modifications of a QD-PUF could be implemented into devices. When fabricated into a PUF, a protective covering is required, to prevent damage, or degradation of the sample. A cover of a solid immersion lens (SIL) will both prevent damage to the sample, but can also help to extract more useful light from the device^33^. Tags such as these can be any size required in each use case, but existing solutions include embedding the nanoparticles within a 3D object, or within a device-recognisable feature. We propose embedding the useful area of the tag within a QR code, allowing for challenges of the tag to be subdivided regions for each CRP, or the whole tag could generate a CRP^34^.

Far-field measurement of tags of optical nanomaterials for security purposes has been discussed in existing literature, including constellation mapping^35^, or spatially-dependent division of the tag into individual measurement points for individual spectra^36,37^. Nanoparticle PUFs based on inks with random pinning points have been measured with fluorescence microscopy^38,39^ and smartphone cameras^19^.

Further enhancements to the quality of the measurement of the tag could utilise the relative intensity of each of the measurements to each other. These points could add another dimension of security to the tag, to measure the relative intensity of each peak, in combination with the hotspots.

## Conclusion

In this work a simple fabrication method was introduced to create a light-emitting plasmonic nanostructure system, with strong spatial intensity variation. The structure used was based on a random distribution of both Au NPs on an Al back-coating with InP/ZnS quantum dots, which couple to enhance PL emission. This structure, with the addition of a highly reflective metallic layer results in hotspots, dramatically increasing the range of emission peaks and peak intensity, creating a larger number of CRPs, and making the sample easier to measure with a conventional CMOS sensor^20^. This unpredictable variability has tremendous potential applications in the field of unique identification, where scattering and emission patterns are easy to produce and impractical to replicate. These findings pave the way towards the development of a simple, large-scale and cost-effective means of producing a practical optical PUF platform, suitable for robust anti-counterfeiting purposes.

## Materials

Gold nanoparticles (3 × 10^9^ ml^−1^ in citrate buffer) were purchased from Sigma-Aldrich. The average diameter of the Au NPs used was 100 nm. The nanoparticles were concentrated with centrifuge and re-dispersed in ethanol before dropcasting. Core/shell InP/ZnS QDs stabilised with oleylamine ligands, concentration 5 mg ml^−1^ were obtained from NN-Labs. All chemicals were used as received from the suppliers.

### Instruments

The morphology and surface density of the QDs and the Au NPs were investigated by Scanning Electron Microscopy (SEM) and Atomic Force Microscopy (AFM). SEM was performed using a JEOL-JSM-7800F with an accelerated voltage of 15 kV. AFM measurements were performed in Peak-Force Mode operation using a Bruker Multimode 5 AFM to characterise the surface of the samples. Silicon nitride tips with a spring constant of 7 Nm^−1^ and a resonant frequency of 140 kHz were used for measurements. PL spectra and PL mapping were recorded in a Horiba LabRAM micro-Raman system using an excitation laser with wavelength of 532 nm. A 100× objective lens, with NA = 0.9 was used for the measurements at ambient temperature giving a laser spot size of approximately 1 μm^2^, and slit width of 200 μm. This was done to help prohibit the influence of interband transitions in Au below around 500 nm, which are assumed to create a different excited electron distribution in the metal NPs^40^. The UV–vis absorption spectra of colloidal QDs and Au NPs were recorded on a Shimadzu UV 3600 spectrophotometer in the range 380–800 nm.

## Supplementary Information

Supplementary Information.
