# Supplementary material for: Hotspot generation for unique identification with nanomaterials
Source: Sci Rep. 2021 Jan 15;11:1528. doi: 10.1038/s41598-020-79644-w (PMC7810830; doi:10.1038/s41598-020-79644-w)
Supplement: Supplementary file 1 — Supplementary Information. [file 41598_2020_79644_MOESM1_ESM.pdf]

Supporting Information

Hot-spot generation for unique identification with nanomaterials

Nema M. Abdelazim[1,2], Matthew J. Fong[1]*, Thomas McGrath[1], Christopher S. Woodhead[1], Furat A. Al-Saymari[1], Ibrahim Ethem Bagci[2], A.T.Jones[1] , Xintai Wang[1,4], Robert J. Young[1]*

[1] Physics Department, Lancaster University, Lancaster, LA1 4YB, UK

[2] School of Electronics and Computer Science, University of Southampton, Southampton, SO17 1BJ, UK

[3] School of Computing and Communications, Lancaster University, Lancaster, LA1 4WA, UK

[4] Cavendish Laboratory, University of Cambridge, J J Thomson Avenue, Cambridge, CB3 0HE, UK

*Corresponding authors: Matthew J. Fong, Email; j.fong@lancaster.ac.uk, Robert J. Young, Email; r.j.young@lancaster.ac.uk

Section 1. Optimising the thickness of spacer layer

Section 2. Preparation of gold nanoparticle films

Section 3. QD film coverage and morphology

Section 4. PL spectra for the control sample

Section 5. PL intensity quenching by effect of Au NP aggregation

Section 1

Optimising the thickness of spacer layer

Direct contact of the QDs with Au NPs will lead to PL quenching from the QDs[1]. The thickness of the dielectric spacer layer between metal and QDs plays an important role in improving the maximum PL hot spot generation.

After the Au NPs were deposited onto the substrates, a controllable insulator spacing layer is deposited to minimise quenching by metal cores. A few-nanometre spacer layer of titanium (Ti) was deposited using an electron-beam evaporator, and then exposed to air under annealing for 2 h to form TiO_2. Ti was deposited at 1.0 Å/s using 75 mA beam current. The e-gun voltage was 10 kV, the chamber base pressure $2x10^{-7}$ mBar and the deposition pressure $1x10^{-6}$ mBar. The layer thicknesses were optimised through both PL measurements and atomic force microscopy (AFM).

It is interesting to find that the optimal enhancement ratios derived from PL characterizations show different thickness dependences on SiO_2 shell. The hot spot enhancement performance is mainly changing by adjusting the thickness of the spacing layer. The thickness of Ti spacer layer was optimised to be about 6 nm for maximised PL. When the TiO_2 spacer is further increased, the PL hot spot intensity is decreased. Maximum enhancement of 5.8 times of the hot spots is achieved at the 6 nm spacer as shown in Figure S1.

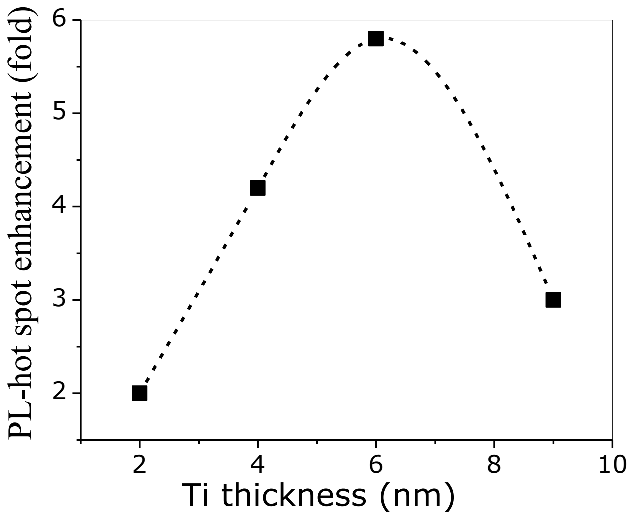

Figure S1. Thickness-dependent PL hot spot enhancement.

Section 2

Preparation of gold nanoparticle (Au NP) films

By controlling the concentration of the Au NP colloids, it is possible to control the film coverage, with monitoring both uniform distribution and aggregation effects on substrates. Au NPs in an aqueous buffer solution (with average diameter 100 nm and concentration of $3.8x10^9$ ml^{-1}) were transferred to 4 centrifuge tubes and then centrifuged at 15000 rpm (21380g) for 30 min. The buffer layer supernatant was carefully removed from the tubes and the resulting solid pellet of AuNPs re-dispersed in ethanol with different volumes (200 µl, 100 µl, 50 µl, 10 µl). The samples solution were then sonicated for 20 min and ready for dropcasting. The final concentrations of the Au NPs were: $6.3x10^9$, $9.5x10^9$, $19x10^9$, $38x10^9$, particles/ml, respectively dispersed in ethanol.

The Au NPs were then deposited onto a substrate by drop-casting. 5 µl of Au NPs in ethanol from each tube were dropped onto the substrates and dried in ambient conditions at room temperature for 1 hr, then dried at 80° C in a vacuum oven for 8 hr. Finally, the samples were cleaned using oxygen plasma to remove any remaining organic solvents.

Figure S2 shows a SEM images of the substrate after using drop-casting with different colloidal Au NP concentrations. The surface number density can reach 105 ~ 110 particles per μm^2, revealed by SEM imaging with the concentration of $19x10^9$, particles/ml (Figure. S2c). By increasing the Au NP concentration in solution up to $38x10^9$, particles/ml, aggregation of the Au NPs in the array was also observed from the SEM imaging (Figure. 2d). The distribution of the NPs in the array is uniform and stable, even after plasma treatment. Our deposition method is viable to various metal nanocrystals with different shapes and sizes to form dense arrays of films. Both the area and the quantity of the arrays can be further scaled-up. The well-separated Au NPs allow deposited large-scale arrays to maintain the plasmonic properties of the individual Au nanocrystal.

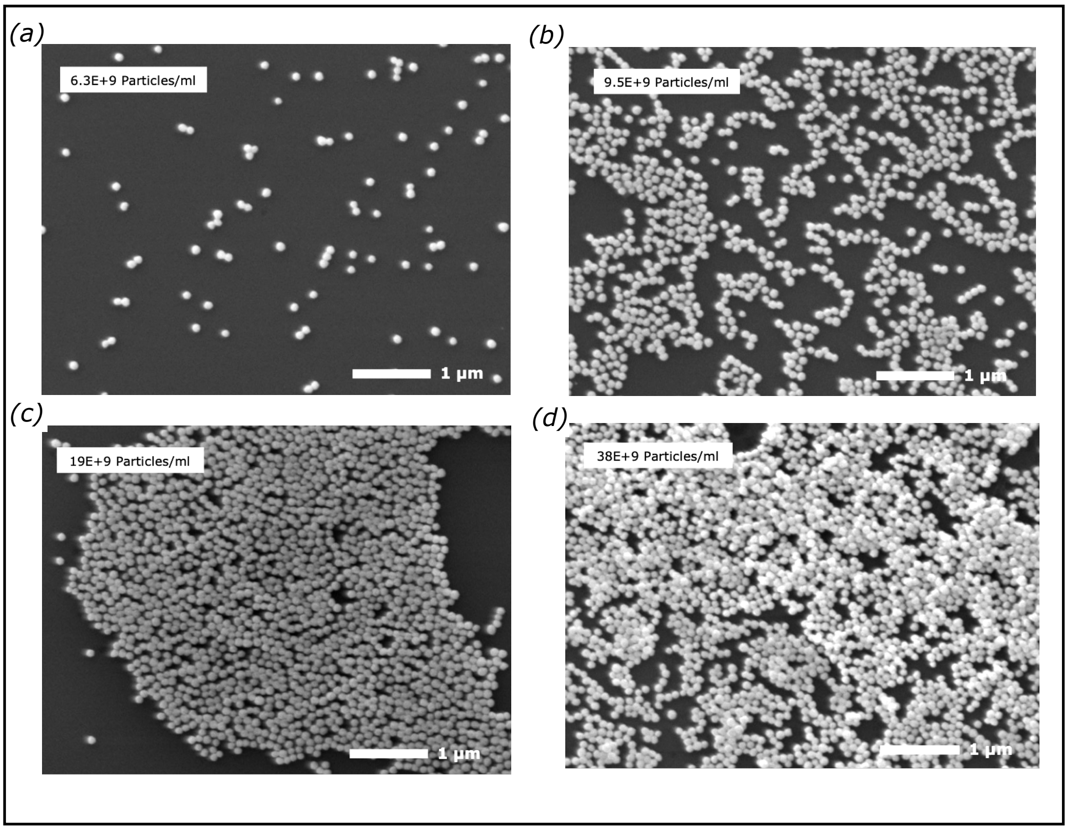

Figure S2. SEM images of deposit different concentrations from colloidal Au NPs on top of SiO_2/Al substrates. Scale bar 1µm.

Section 3

QD film coverage and morphology

To confirm that the PL enhancement arises from the interactions between QDs and Au NPs, and not from any aggregation or cracks on the film surface, it is essential to get full surface coverage and a very uniform QD film. In our study we developed a room temperature spray-coating technique to achieve full surface coverage and uniform films from QDs top layer. The electrospray system used here is consists of: a syringe with a 30-gauge needle which contains the QD solution, a high voltage (1- 10 kV) DC power supply, conducting plate and endoscope as shown in the sketch Figure 3a. The QDs dispersed in toluene (5 mg/ml) were mixed prior to spray with ethanol/toluene (1:0.5:0.5 v/v) and stirred for 20 min. The size of the droplets was carefully adjusted to be each droplet contains one single individual QD, this allows the single QDs to distribute in between the Au NPs. Since the size of the droplets is not only be based on the applied voltage, qualitative parameters deposition such as electrode gap, the density of the mixture and other were determined to optimise the QD film's coverage. The best conditions for the spraying to perform a uniform film on a large area substrate were found to be: flow rates of 0.3-0.5 ml/h, voltages between 3.5 and 4.5 kV and the distance between substrate surface and syringe tip was 8-10 cm. Both plasmonic nanostructure sample and control samples were sprayed simultaneously.

Three different QDs colloidal concentrations were used to optimise the best coverage for a uniform film. For low concentration (0.75 mg/ml) Figure S3b, the QDs do not fully cover the substrate and the film was not uniform. At 1.25 mg/ml the QDs start to fill up the voids, and the film looks more uniform. However, at a concentration of 2.5 mg/ml the substrate is fully covered and aggregations begin to appear. Further increasing the density of the QDs may cause shielding of the effect of the Au NPs, because the QDs should be between the Au NPs, not on the top of it for most efficient PL emission intensity.

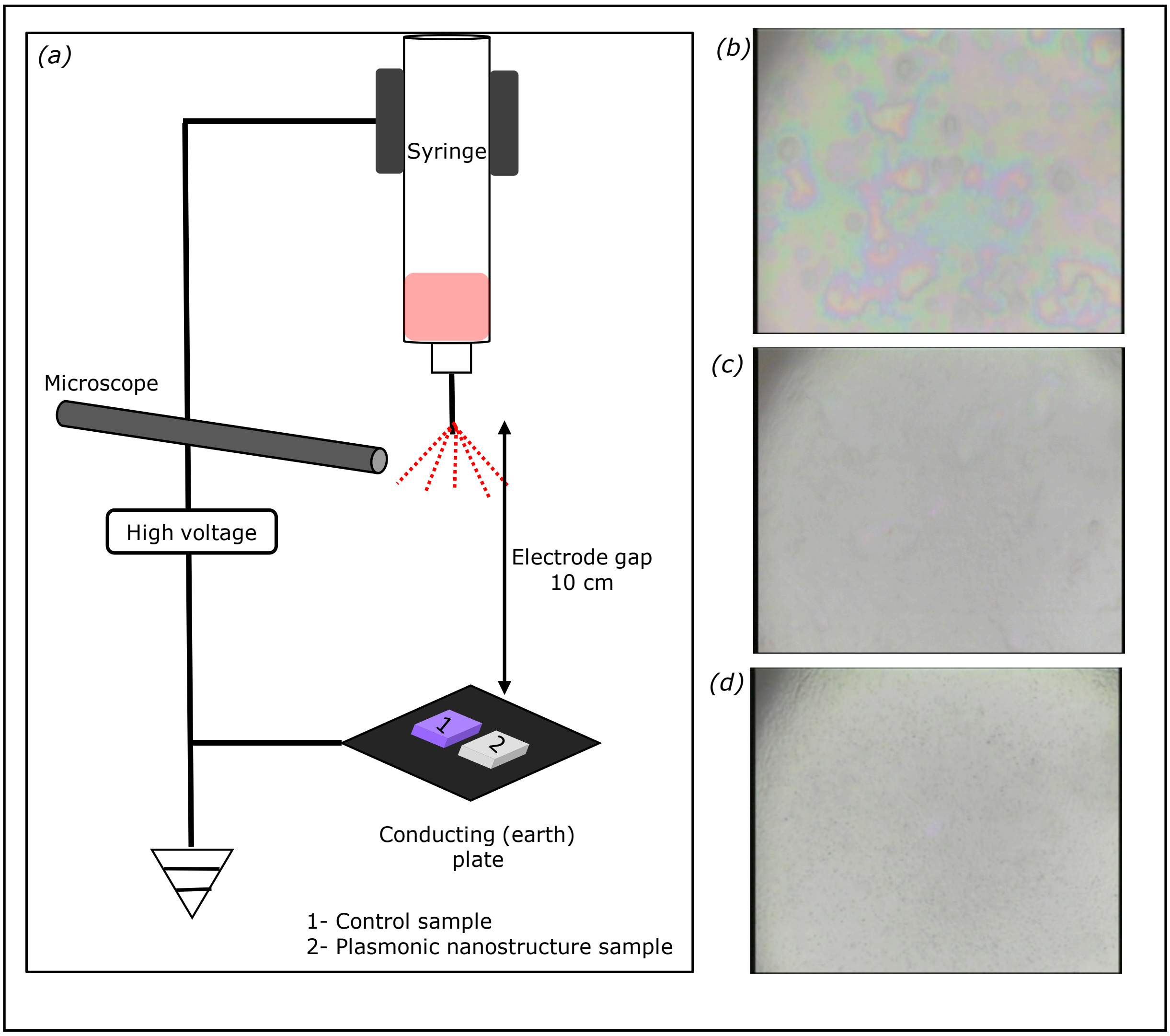

Figure S3. (a) Sketch of the electrospray process. (b-d) The optical microscopy images (x 100) after spray-coating shows how the QD film morphology evolve with the increasing the amount of the QDs from 0.75 mg/ml to 2.5 mg/ml.

Section 4

PL spectra for the control sample

Figure 4 shows PL spectrum of the control sample at different scanning areas. It is observed that the PL intensities and peak wavelength were reasonably uniform, demonstrating no aggregation from QDs after spraying process. All PL intensity values between 1-2 kcounts/sec.

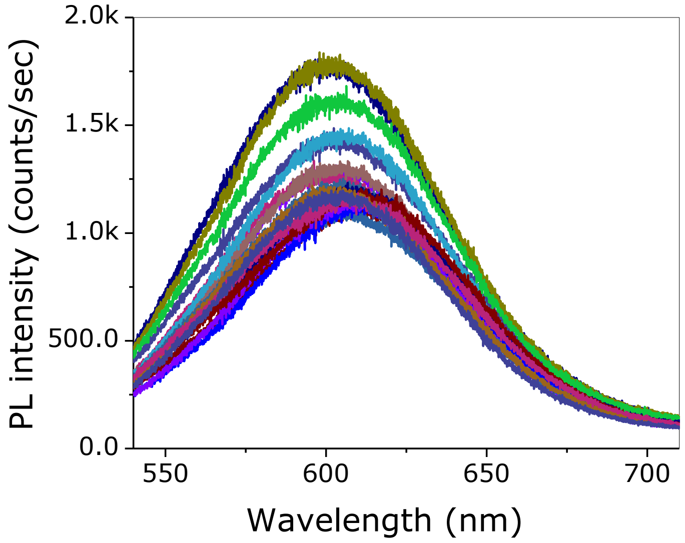

Figure S4. Set of PL emission scan obtained from different positions on the surface of the control sample corresponding to mapping area (A1).

Section 5

PL intensity quenching by effect of Au NPs aggregation

We observed quenching of PL emission of QDs in the presence of Au NPs in some areas (marked mapping area B6), at very small interparticle distances, or due to the nanoparticles' aggregation[2] as shown in SEM Figure S5a. They are a significant feature when comparing the PL spectra from control sample. The PL emission appears to have a different spectral shape under the same excitation conditions, which is due to the different relative contribution of the aggregated and non-aggregated Au NPs [3]. The new PL features became significantly weak, and gradually broadened which shows in some cases two broad bands positioned at 560 nm and 610 nm. The weak additional broad peaks at high energy may be due to Au NPs on an Al-coated film which emit broad band unconverted luminescence near the plasmon resonance wavelength [4]. The emission wavelength of the Au NPs depended on the surface coverage not

on Au NPs size [5], thus we can see different shapes from PL spectrum as shown in Figure 5b. Both enhancing and quenching effects are also sensitive to the distance between the fluorescent (i.e QDs) and the metal nanoparticle. A broad spectral feature of the emission peaks with FWHM up to 130 nm that can be attributed to a higher aggregation at scanning area B6.

In a non-uniform distribution, Au NPs clustering or aggregation areas prevents the QDs from being distributed between the metal particles, resulting non-embedded structures, which may cause quenching.

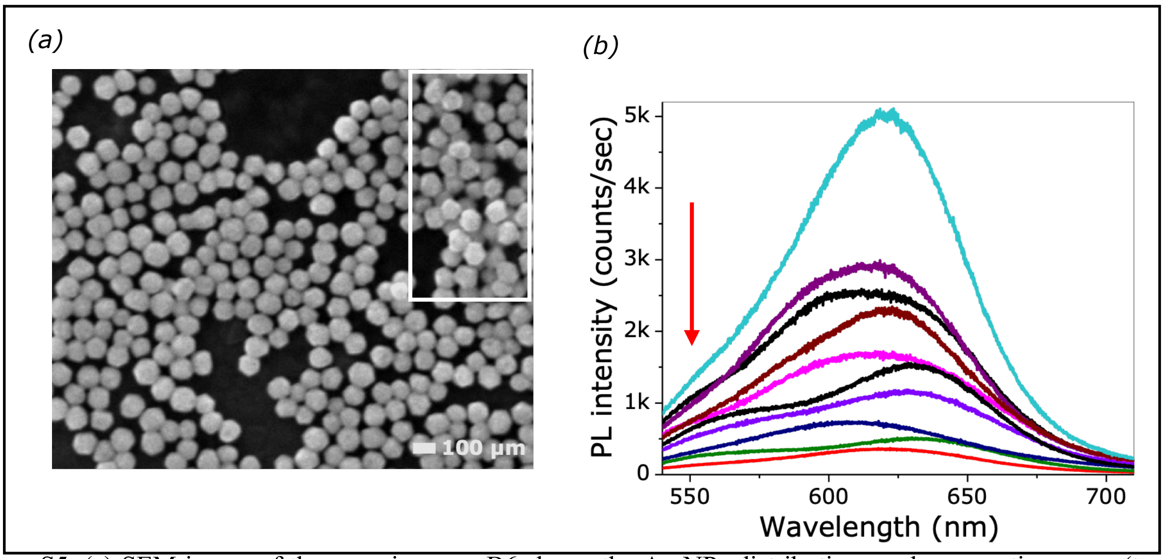

Figure S5. (a) SEM image of the scanning area B6 shows the Au NPs distributions and aggregations area (top right), (b) Set of PL emission scan obtained at same location from where the SEM image was taken. The red arrow indicates the second broad spectral peak arising from the cluster of AuNPs.

References

1. Geddes, C. D., *Surface plasmon enhanced, coupled and controlled fluorescence*. John Wiley & Sons: 2017.
2. Haridas, M.; Tripathi, L.; Basu, J. *Applied Physics Letters* **2011,** 98, (6), 27.
3. Abdellatif, M. H.; Salerno, M.; Abdelrasoul, G. N.; Liakos, I.; Scarpellini, A.; Marras, S.; Diaspro, A. *Beilstein journal of nanotechnology* **2016,** 7, (1), 2013-2022.
4. Lumdee, C.; Yun, B.; Kik, P. G. *Acs Photonics* **2014,** 1, (11), 1224-1230.
5. Liu, J.; Duchesne, P. N.; Yu, M.; Jiang, X.; Ning, X.; Vinluan III, R. D.; Zhang, P.; Zheng, J. *Angewandte Chemie International Edition* **2016,** 55, (31), 8894-8898.
